# Introducing the “Bone-Screw-Fastener” for improved screw fixation in orthopedic surgery: a revolutionary paradigm shift?

**DOI:** 10.1186/s13037-017-0121-5

**Published:** 2017-03-20

**Authors:** Philip F. Stahel, Nicholas A. Alfonso, Corey Henderson, Todd Baldini

**Affiliations:** 1 Department of Orthopaedic Surgery, Denver Health Medical Center, Denver, CO 80204 USA; 2 Department of Orthopaedic Surgery, University of Colorado School of Medicine, Aurora, CO 80045 USA

## Abstract

**Background:**

Conventional screws used for fracture fixation in orthopedic surgery continue to rely on the historic buttress thread design. While buttress screws generally provide solid resistance against unidirectional axial loading forces, their design suffers from several limitations, as the buttress thread does not adequately resist multiaxial forces. Furthermore, the buttress screw is prone to stripping at the bone-screw interface and can cause microfracturing of the surrounding bone due to its thread design. Standard buttress screws are therefore at risk of adverse postoperative outcomes secondary to failure of bone fixation. A new patented Bone-Screw-Fastener was recently designed that is based on an interlocking thread technology. This new fastener provides distributive forces from the threads onto the bone and therefore resists loads in multiple directions. The underlying concept is represented by a “female thread” bone cutting technology designed to maximize bone volume, preserve bone architecture, and create a circumferential interlocking interface between the implant and bone that protects the thread from stripping and from failing to multiaxial forces.

**Presentation of the hypothesis:**

We hypothesize that the new Bone-Screw-Fastener overcomes the classic shortcomings of conventional orthopedic screws with buttress threads by ease of insertion, improved bone preservation, increased resistance to off-axis multidirectional loading forces and to stripping of the threads. These advanced biomechanical and biological properties can potentially mitigate the classic limitations of conventional buttress screws by providing better resistance to implant failure under physiological loads, preserving bone biology, and thus potentially improving patient outcomes in the future.

**Testing the hypothesis:**

The presumed superiority of the new fastener will require testing and validation in well-designed prospective multicenter randomized controlled trials (RCTs), using the conventional buttress screw as control.

**Implications of the hypothesis:**

Once validated in multicenter RCTs, the new Bone-Screw-Fastener may drive a change in paradigm with regard to its innovative biomechanical principles and biologic bone preservation for surgical applications requiring screw fixation.

## Background: a brief history of bone screw design

**Fig. 1:**
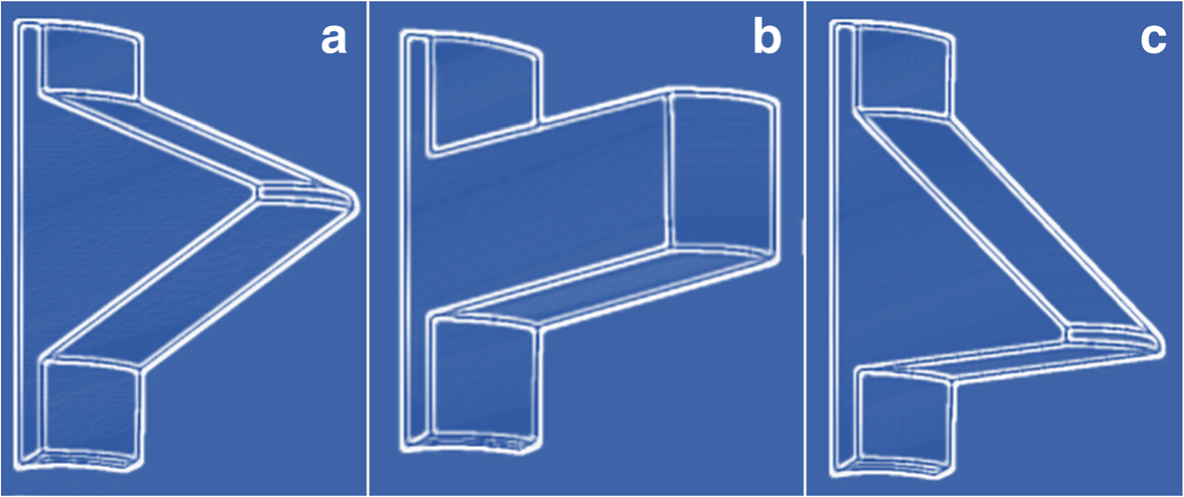
The historically prevalent screw thread shapes include the V-shaped thread (**a**), the square thread (**b**), and the buttress thread (**c**). The buttress thread represents the current paradigm of screw design in orthopedics due to improved unidirectional pull-out resistance in bone

Archimedes of Syracuse (287-212 BC) is considered the inventor of the first screw in ancient times [1]. His invention was initially designed to remove the bilge water from large ships using a water-pump based on a revolving screw-shaped blade inside a cylinder [1]. Archimedes’ screw principle is still in use today for pumping water and transporting coal or grain. Interestingly, the introduction of industrial screws was delayed by a thousand years after Archimedes’ invention, due to technical challenges in screw manufacturing [2]. In 1850, screws were applied for the first time in orthopedic surgery by the French surgeons Cucel and Rigaud, who used two wood screws and a leather strap to fixate an olecranon fracture [3]. In the early 20^th^ century, William O’Neill Sherman (1880–1979) was a pioneer of internal fracture fixation who modified conventional screw designs to orthopedic applications [4]. Of note, Sherman’s screw design remained the “gold standard” in orthopedics until the introduction of the AO screw half a century later [5]. Stainless steel was introduced in the 1920s and allowed better biocompatibility of bone screws [5]. In the 1940s, the Belgian surgeon Robert Danis (the “father of modern osteosynthesis”) further modified screw designs to applications specific to human bone by implementing the following three technical features [6]:

1. A change of the ratio from the exterior screw diameter to core diameter from 4:3 in industry metal screws, to 3:2 in orthopedic screws;
2. A reduction of thread surface area to 1/6, based on the notion that bone strength is about 1/6 of the strength of metal;
3. A change from the classic industrial V-shaped thread design to buttress threads (Fig. 1), based on the postulated increased pull-out resistance of buttress threads.

Robert Danis’ pioneering work on internal fixation, including improved screw design and plate technology, preceded the foundation of the AO (“Arbeitsgemeinschaft für Osteosynthesefragen”) in 1958 in Switzerland [6, 7]. One of the fundamental subsequent achievements of the AO was the global standardization of surgical principles and techniques, and the introduction of a uniform design for orthopedic implants and instruments [5].

## Principles of screw threads

Screw threads are designed to optimize initial contact and surface area, dissipate and distribute stress forces at the screw-bone interface, and increase pull-out resistance to load [8]. The basic principles of screw thread geometry include thread shape, face angle, pitch, depth, and width. Thread pitch, depth and width are highly variable among the available orthopedic screws on the market. For example, cancellous screws have an increased thread depth compared to cortical screws, with the intent of increasing the surface area for improved screw purchase in lesser quality bone [9]. Thread pitch refers to the linear distance travelled by the screw after one full turn. In implants with equal length, a smaller pitch implies a higher number of threads. For example, locking head screws have a lower pitch than conventional cortical screws. Among the multiplicity of thread shapes, the buttress thread design remains the historic paradigm for the shape of current orthopedic screws. From the perspective of the face angle of buttress screws, the load-bearing face is typically perpendicular (or inclined up to 5-7°) to the axis of the screw, whereas the other face of the thread is angled at around 45° (Fig. 1c). The popularity of buttress threads in current orthopedic screw designs is reflected by the advantage of handling high axial thrust in one direction which leads to increased shear strength and improved unidirectional pull-out resistance compared to other conventional thread shapes [10–12]. However, orthopedic screws are typically not challenged by axial loading forces from physiological motion in vivo. Thus, standard buttress screws remain at a significant risk of failure when exposed to multidirectional loading forces [13]. In an attempt to address the physiological multiaxial loading environment, newer generation locking plates have been able to reduce the risk of implant failure, particularly in osteoporotic bone [14]. Locked plating technology relies on the benefit of a fixed-angle construct that does not rely on friction and compression forces between implant and bone. However, locking head screws have been shown to have their own set of shortcomings [14], including the stiffness of plate-screw constructs and increased cost, hence research continues towards more effective and equitable, cost-conscientious solutions to failures at the bone-implant interface.

In essence, until present, the historic buttress screw continues to represent the main pillar in orthopedic screw design, despite significant biomechanical shortcomings associated with high failure rates [13, 15].

## Limitations of conventional buttress screws

Most of the currently applied screws in orthopedic surgery utilize a form of the buttress thread [5, 6]. However, the buttress thread suffers from several intrinsic limitations. The screws are hard to start within the bone interface. Buttress screws can miss the far cortex through the projected trajectory during insertion, which may lead to stripping out the near cortex. Even with adequate insertion, during the final screw tightening, the axial load and torque applied may overcome the bone resistance, which then results in the buttress screw stripping out of both cortices. In addition, as the buttress thread induces a radial force that is perpendicular to the screw’s long axis, this increases the probability of creating a stress riser or an incidental fracture to the adjacent bone bridge. Traditional buttress threads are designed to resist unidirectional axial loads only. However, the physiological in vivo loading on orthopedic implants is known to be multiaxial and can, therefore, result in loosening. One manifestation of buttress thread failure is screw loosening and “toggling” which entails that the screw erodes through the bone and enlarges the hole within which the screw resides, which may lead to failure of fixation. In clinical application, orthopedic screws must resist dynamic forces generated during patients’ daily activities. Unfortunately, current buttress screws are not designed to resist multidirectional force, which increases the risk of postoperative complications, including screw loosening and failure of fixation. Since the selected modifiable variables of buttress screw designs (thread pitch, depth, width and face angle) are interrelated, attempts to improve screw retention will increase the amount of friction and insertion torque, thus resulting in heat generation and potential heat necrosis to the adjacent bone. Excess heat during screw insertion will compromise the screw’s purchase and retention of the thread interface at the site of necrotic bone. The conventional buttress cutting mechanism generally does not provide precise thread forming and therefore represent more of a “rough” cutting tool that leads to microfracturing of the bone around the threads. This bone debris accumulates along the thread teeth and increases insertion torque and friction which generates additional heat. The debris also makes the screw harder to insert and provides a poor interface between screw and bone. The “rough” or imprecise cutting mechanism of buttress threads is thought to represent one of the underlying root causes of bone-implant failures resulting from high compressive forces, increased insertion friction with heat generation, and presence of bone microfractures within the threads. All of these factors contribute to the risk of bone necrosis or bone resorption around the screw.

In summary, the essential shortcomings of buttress threads include the risk of stripping, screw loosening, induction of stress risers, bone microfracturing, heat necrosis, with subsequent failure of fixation and risk of creating fracture nonunions and malunions. These fundamental problems have remained unaddressed in past efforts aimed at improving the design of modern orthopedic screws due to the ongoing reliance on the conventional historic buttress thread concept.

## The hypothesis

We hypothesize that the new Bone-Screw-Fastener overcomes the classic shortcomings of conventional orthopedic screws with buttress threads by ease of insertion, improved bone preservation, increased pull-out resistance to multidirectional loading forces and resistance to stripping of the threads. These advanced biomechanical and biological properties may mitigate the classic limitations of conventional buttress screws by providing better resistance to implant failure under physiological loads, preserving bone biology, and thus, improving patient outcomes in the future.

## Presumptive advantages of the new fastener design

A new Bone-Screw-Fastener was designed based on an interlocking bone-implant interface technology that provides a distribution of forces from the implant onto the bone and subsequently resists loads in all directions. The new fastener consists of a “female thread” bone cutting technology designed to maximize bone volume, preserve bone architecture, and create a circumferential interlocking interface between the implant and bone, similar to a “nut-and-bolt” technology. The following intuitive advantages by the Bone-Screw-Fastener support the hypothesis of superiority to conventional orthopedic screws with buttress threads:

1. The new interlocking thread pattern is designed to resist multidirectional forces and bending moments to limit the toggling of the implant and minimize radial forces. These properties provide improved resistance to failure and decreased risk of creating stress risers and iatrogenic fractures to adjacent bone.
2. The interlocking thread pattern is designed to allow for higher finishing torque values compared to implants with buttress threads, and to resist screw stripping, even in lesser quality bone.
3. The new bone cutting mechanism is designed to curl the bone chips away from the cutting edges to create a solid bone-implant interface free of debris and to prevent iatrogenic bone destruction during screw insertion.

**Fig. 2:**
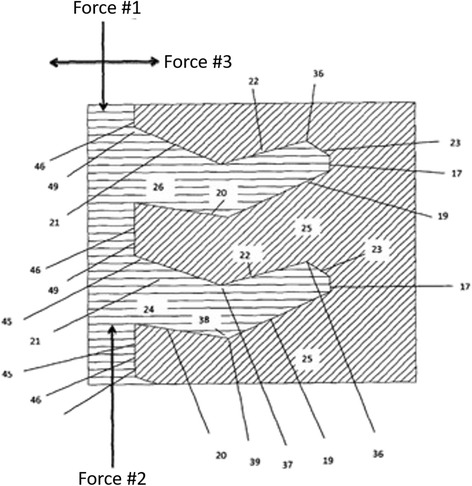
Cross-section of the new Bone-Screw-Fastener thread configuration, with reference points to different loading forces. See text for details and explanations

**Fig. 3:**
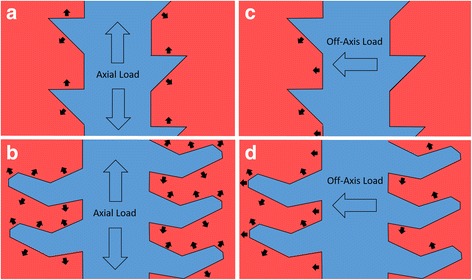
Comparison of load vectors on the threads of the new Bone-Screw-Fastener compared to the conventional buttress thread resulting from an *axial* loading force (**a**, **b**) and from an *off-axis* loading force (**c**, **d**)

As demonstrated in the schematic drawing in Fig. 2, the various reference points operate in pairs such that the intersection defines centering points for load distribution and force equalization (e.g. points 19/20 and 21/22, respectively). Furthermore, an axial loading force #1 applied on the fastener induces centering point 38 unto 39, thus embedding the screw onto the bone. Likewise, when force #2 is applied, centering points 36 and 37 are embedded onto each other, preventing movements upon application of a horizontal force #3. The new fastener is designed to provide greater retention of bone structure, and the bone tooth volume (#25 in Fig. 2) can be optimized to adapt to varying physiological conditions and to specific anatomic locations and differing bone quality. The fastener threads allow for improved distribution of forces across the multiple thread faces after axial loading (Fig. 3b), compared to a standard screw with buttress threads (Fig. 3a). This important biomechanical property particularly applies to off-axis loading scenarios, where force distribution is significantly improved in the fastener (Fig. 3d) compared to the conventional buttress screw (Fig. 3c).

**Fig. 4:**
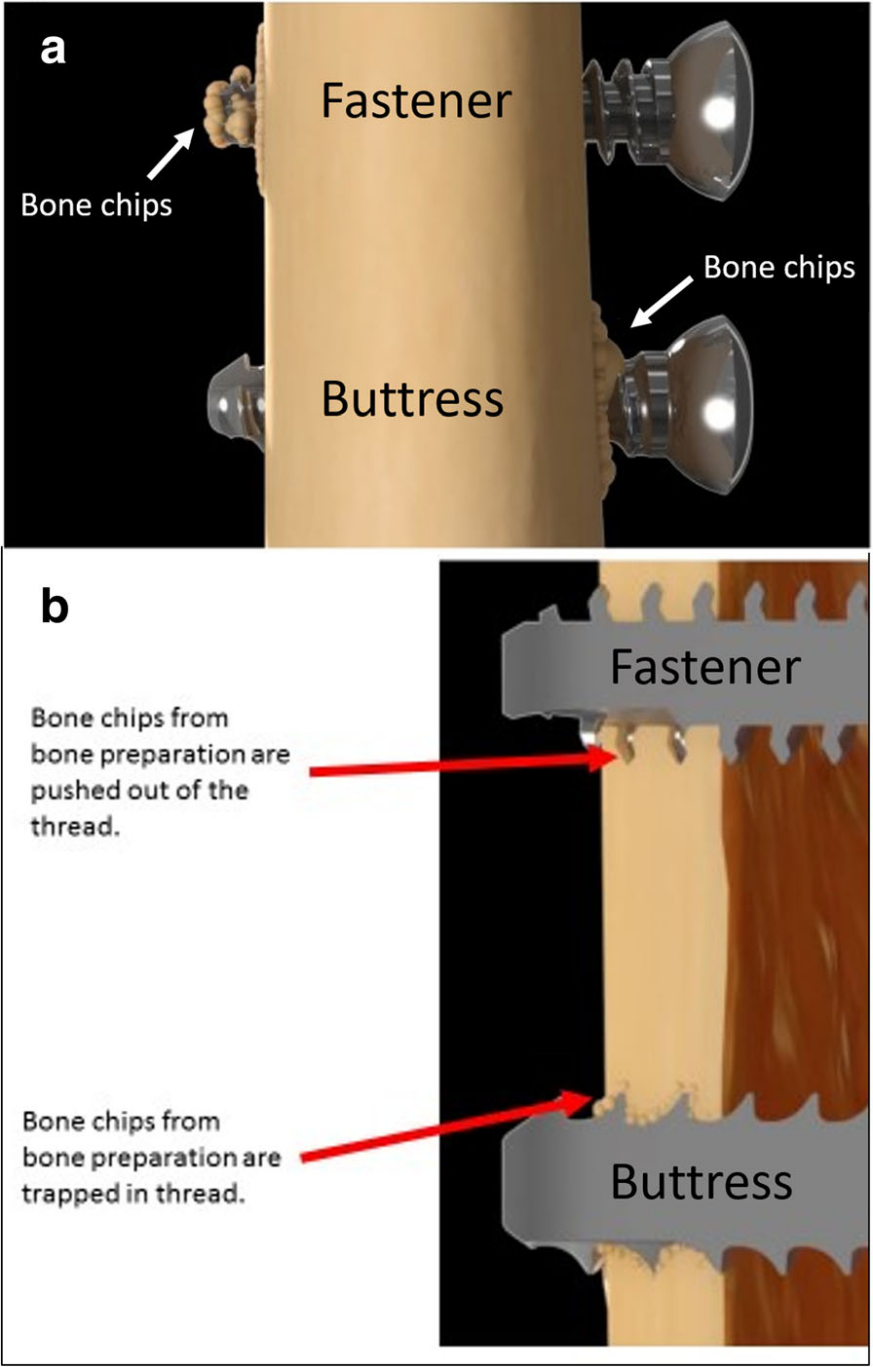
Comparison of bone debris location (“bone chips”) during implant insertion between the new Bone-Screw-Fastener and the conventional buttress screw. Panel **b** represents a cross-section of panel **a**

Finally, an additional relevant benefit of the new Bone-Screw-Fastener is bone preservation. The fastener’s new thread cutting mechanism provides a superior thread forming tool that prepares the bone for implant placement by cutting precise “female threads” into the bone. With the new interlocking thread technology, the cut bone chips curl away from the cutting edges of the fastener, and are fed forward into the predrilled pilot hole (Fig. 4a). The underlying mechanism relies on the flute being “left-handed” on a right-handed threaded fastener. In other words, as the fastener advances, the flute forces the bone chips forward into the pilot hole ahead of the screw. This cutting mechanism results in bone clearance and formation of “bone teeth” in the tissue that engages the threaded fastener (Fig. 4b). The interface between the bone and fastener is then free of the cuttings, provides “healthier” bone tissue adjacent the fastener, and prevents iatrogenic microfracturing of the bone, as seen with the use of standard buttress threads (Fig. 4b).

## Clinical pilot series

The new Bone-Screw-Fastener (SMV Scientific, Austin, TX) received FDA clearance on June 23, 2015, for clinical use in 510(k) #K150981 for the following indications:

*“The SMV Scientific 3.5mm and 4.0mm Bone Screws are intended for fixation of fractures, osteotomies and non-unions of the clavicle, scapula, olecranon, humerus, radius, ulna, pelvis, tibia, calcaneus, femur and fibula in adults and in both children (2–12 years) and adolescents (12–21 years) in which growth plates have fused or in which growth plates will not be crossed by screw fixation”.*

**Fig. 5:**
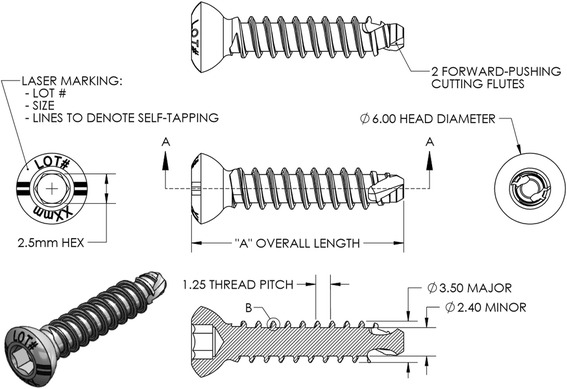
Schematic presentation of the structure and dimensions a self-tapping 3.5mm cortical Bone-Screw-Fastener

**Fig. 6:**
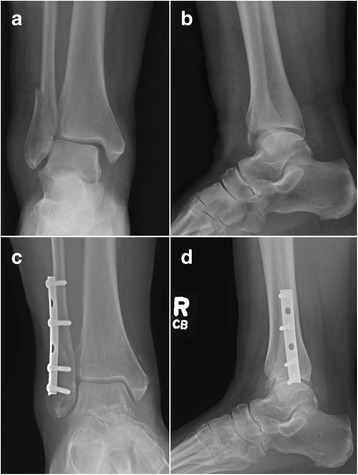
Case example of a 65 year-old female patient who sustained an unstable SER-4 equivalent right ankle fracture (**a**, **b**). The injury was managed by open reduction with internal fixation of the lateral malleolar fracture using a posterolateral antiglide plate and four pretapped Bone-Screw-Fasteners. The fracture healed uneventfully in anatomic position, as demonstrated on 6 months follow-up radiographs (**c**, **d**)

The fastener was approved for clinical use at Denver Health, the regional academic level 1 trauma center and safety-net hospital in Colorado, in July 2015. The 3.5mm fastener was subsequently used in selected surgical applications per standard of care as a substitute to standard 3.5mm cortical buttress screws, 4.0mm cancellous bone screws, and 3.5mm locking plate constructs. The fastener was either used as an independent lag screw or positioning screw, or in conjunction with small-fragment stainless steel plates. During a time-window from August 24, 2015, until December 31, 2016, the first author (P.F.S.) performed 30 surgical procedures in 30 patients that included implantation of at least one Bone-Screw-Fastener. Surgical indications were placed by standard of care in all patients. During the initial pilot phase until January 31, 2016, the fasteners required pre-tapping, whereas self-tapping fasteners were introduced on February 1, 2016, and used exclusively thereafter. For illustration, Fig. 5 demonstrates a schematic drawing of the self-tapping fastener thread configuration. The retrospective analysis of this observational cohort study was approved by the Colorado Multiple Institutional Review Board (COMIRB) at the University of Colorado (Protocol # 16-0297). The study was determined to meet criteria for full waiver of consent due to the retrospective study design. One 10-year old patient with a displaced Salter-Harris III type ankle injury was excluded from analysis per study protocol as a minor of less than 18 years of age. The remaining 29 patients were included in the retrospective observational cohort analysis. These 29 patients had a total of 123 Bone-Screw-Fasteners implanted during the observational study time-window. Of these, 84 fasteners required pre-tapping, and 39 fasteners were self-tapping. All fasteners used in this study were 3.5mm stainless steel implants. The first 3.5mm Bone-Screw-Fastener was applied as part of a bimalleolar ankle fracture fixation in a 24 year-old male patient on August 24, 2015. The patient demographics, fracture classification, and respective procedures performed are shown in Table 1. All patients followed up for a minimum of 3 months, with an average follow-up time of 10 months (±3.5 SD; range 3–15 months). There were no intraoperative or postoperative complications in the 29 patients included in this pilot series. No technical problems were noted with insertion of the fasteners, and no radiographic signs of implant loosening were noted in any of the 29 patients. All fractures healed clinically and radiographically within an average of 3 months of follow-up. Figure 6 demonstrates a representative example of a 65 year-old lady whose unstable SER-4 equivalent ankle fracture was shown to be healed in anatomic position at 6 months follow-up after fixation by an antiglide plate construct with four Bone-Screw-Fasteners. Six patients (20.7%) required a return to the operating room for removal of symptomatic implants. Of these, 5 patients had healed ankle fractures with symptomatic plates around the distal fibula, and one patient required a plate removal after temporary bridging of the acromioclavicular joint (Table 1). No technical problems were noted with removal of the 21 fasteners that were removed in these six patients at an average of 9.5 months (± 3.5 SD; range 5–15 months).

**Table 1:** Demographic data of the 29 patients included in the pilot study^a^

Patient No. (Gender/Age)	AO fracture classification	Procedure performed	Follow-up	Surgical revision (time)
1 (M/27)	44-B3.2	ORIF ankle Fx	1 year	Implant removal (8 months)
2 (M/41)	44-B2.1	ORIF ankle Fx	1 year	None
3 (M/68)	61-B2.2	ORIF pelvic Fx	1 year	None
4 (F/39)	44-C2.3	ORIF ankle Fx	1 year	None
5 (M/24)	44-B3.1	ORIF ankle Fx	1 year	None
6 (F/24)	44-C2.3	ORIF ankle Fx	15 months	Implant removal (15 months)
7 (F/53)	11-B2.1	ORIF humerus Fx	1 year	None
8 (M/45)	44-C2.3	ORIF ankle Fx	1 year	None
9 (M/60)	10-B3.3	AC reconstruction	1 year	Implant removal (5 months)
10 (M/23)	15-B2.3	ORIF clavicle Fx	1 year	None
11 (F/35)	44-A3.3	ORIF ankle Fx	1 year	None
12 (M/24)	44-B2.1	ORIF ankle Fx	1 year	None
13 (F/33)	44-B3.3	ORIF ankle Fx	1 year	Implant removal (9 months)
14 (M/23)	44-B2.3	ORIF ankle Fx	1 year	None
15 (M/50)	23-C2.1	ORIF distal radius Fx	1 year	Implant removal (12 months)
16 (F/65)	44-B2.1	ORIF ankle Fx	1 year	None
17 (F/61)	44-B2.1	ORIF ankle Fx	1 year	None
18 (F/44)	44-B3.1	ORIF ankle Fx	1 year	None
19 (F/27)	44-C2.3	ORIF ankle Fx	1 year	None
20 (F/77)	44-B2.1	ORIF ankle Fx	1 year	None
21 (F/58)	44-B2.1	ORIF ankle Fx	9 months	None
22 (F/62)	44-B2.2	ORIF ankle Fx	9 months	Implant removal (8 months)
23 (M/25)	43-C3.2	ORIF tibial pilon Fx	6 months	None
24 (M/53)	44-B3.3	ORIF ankle Fx	6 months	None
25 (F/68)	44-B3.2	ORIF ankle Fx	6 months	None
26 (M/32)	44-B3.1	ORIF ankle Fx	3 months	None
27 (F/18)	44-B3.2	ORIF ankle Fx	3 months	None
28 (F/68)	44-B3.3	ORIF ankle Fx	3 months	None
29 (M/29)	43-B3.2	ORIF tibial pilon Fx	3 months	None

^a^Abbreviations: *AC* acromioclavicular joint, *AO* Arbeitsgemeinschaft für Osteosynthesefragen, *Fx* fracture, *ORIF* open reduction and internal fixation

## Testing the hypothesis

The intuitive biomechanical and biological advantages of the new Bone-Screw-Fastener, in conjunction with the safety and feasibility for clinical application demonstrated in our early experience in 29 patients, provide the rationale for future testing of the fastener in well-designed prospective and controlled studies. Ideally, these studies should be designed as multicenter randomized controlled trials (RCTs), with the conventional buttress screw serving as the control group. The a-priori null hypothesis may postulate equality between the new fastener and the conventional buttress screw in retaining surgical fixation in a specified subset of frequent fractures amenable to screw and plate fixation per standard of care. Rejection of the null hypothesis may prove superiority of the fastener based on defined primary and secondary outcome measures, including failure of fracture fixation, necessity of unplanned surgical revisions, incidence of malunions and nonunions, and patient-reported outcome metrics, using the patient-reported outcomes measurement information system (PROMIS). The methodology should include concealed allocation to treatment cohorts, intention-to-treat analyses, and apply to general requirements by the CONSORT statement [16].

## Implications of the hypothesis

Based on the intrinsic limitations and historic shortcomings of the conventional buttress screw in fracture care, proving superiority of the new patented Bone-Screw-Fastener in well-designed future RCTs may drive a change in paradigm in screw technology. It is conceivable that the insights from orthopedic fracture care may be safely extrapolated to other indications, including maxillofacial surgery, dental implants, spine surgery, joint replacement surgery, and sports surgery, to name a few intuitive surgical disciplines. As our initial pilot study was purely observational and exclusively designed as a “proof of concept” safety and feasibility study, the superiority of the new fastener over standard buttress screws requires validation in future high-quality RCTs.
